# Batch Fabrication of Silicon Nanometer Tip Using Isotropic Inductively Coupled Plasma Etching

**DOI:** 10.3390/mi11070638

**Published:** 2020-06-29

**Authors:** Lihao Wang, Meijie Liu, Junyuan Zhao, Jicong Zhao, Yinfang Zhu, Jinling Yang, Fuhua Yang

**Affiliations:** 1 Institute of Semiconductors, Chinese Academy of Sciences, Beijing 100083, China; lhwang@semi.ac.cn (L.W.); mjliu@semi.ac.cn (M.L.); junyuanzhao@semi.ac.cn (J.Z.); fhyang@semi.ac.cn (F.Y.); 2 Center of Materials Science and Optoelectronics Engineering, University of Chinese Academy of Sciences, Beijing 100049, China; 3 Jiangsu Key Laboratory of ASIC Design, Nantong University, Nantong 226019, China; jczhao@ntu.edu.cn; 4 State Key Laboratory of Transducer Technology, Shanghai 200050, China

**Keywords:** inductively coupled plasma etching, nanometer tip, high aspect ratio, small apex

## Abstract

This work reports a batch fabrication process for silicon nanometer tip based on isotropic inductively coupled plasma (ICP) etching technology. The silicon tips with nanometer apex and small surface roughness are produced at wafer-level with good etching homogeneity and repeatability. An ICP etching routine is developed to make silicon tips with apex radius less than 5 nm, aspect ratio greater than 5 at a tip height of 200 nm, and tip height more than 10 μm, and high fabrication yield is achieved by mask compensation and precisely controlling lateral etch depth, which is significant for large-scale manufacturing.

## 1. Introduction

Nanometer tips, as core components of scanning probe microscopy (SPM) probes, field emission tips, microneedle arrays, etc., are widely used in material surface analysis, bio-engineering, high density data storage and micro-processing [1,2,3,4,5,6,7,8,9,10,11,12,13]. The height, aspect ratio, and radius of the tip are critical parameters which have a significant impact on the tip performance. The tips with small apex radius, high aspect ratio, and large height can achieve high scanning resolution and accuracy in SPM systems [14,15,16,17]. However, Si nanometer tips are apt to wear and have short lifetimes, which results in high use cost, therefore, nanometer tips with long lifetime and low cost are highly desired. 

Several fabrication methods for nanometer tips have been developed, such as tip growing [18,19,20,21,22,23], back-filling [24,25], and tip etching. Depositing materials directly onto a cantilever or a pyramid using vapor-liquid-solid (VLS), focused ion beam (FIB) or focused electron-beam-induced deposition can realize fine needle tips, but the cost for individual growth is too high and fabrication process is too long, which limits the scale of production. The back-filling technique involves etching a tip-like groove on a substrate firstly and then depositing a thin film to obtain a hollow tip. It can make tips from various functional materials at a wafer level, but it is hard to achieve tips with high aspect ratio and small apex radius. Wet or dry etching routines are often used for tip fabrication. Wet etching process for Si tips using tetramethylammonium hydroxide (TMAH), KOH, or HF: HNO_3_: CH_3_COOH mixed solutions is simple and low-cost [26,27,28,29,30,31]. However, wet etching using TMAH and KOH has a high crystal orientation dependency and requires extremely precise alignment with the mask [28,29]. In the HF: HNO_3_: CH_3_COOH etching method it is difficult to control the process and maintain a stabilized etch rate [31]. 

In contrast, dry etching process based on SF_6_, XeF_2_, and other gases can be precisely controlled by adjusting the gas flow rate, etching power, chamber pressure, and so on. Different dry etching approaches have been reported to achieve Si tips for various applications, for example, an isotropic dry etching process for tips with small height (3.6 μm) and large apex (25–40 nm) [32,33,34,35], a multi-step etching approach for “rocket tips” with height greater than 10 μm [36,37,38], the design of ultra-small masks for tips with aspect ratios larger than 5 but small height (less than 3 μm) [39,40]. However, all these approaches have difficulties in batch fabrication of high-end tips due to the complicated process, the strict mask preparation, and low yield. In this work, batch fabricated tips with high aspect ratio, small apex radius, and large height are presented. A simple and reliable fabrication process with excellent etch profile was explored by optimizing the mask pattern and isotropic ICP etching parameters. Mask compensation and precisely controlling the etch procedure showed a dramatic improvement of homogeneity and repeatability, which is a valid method for large-scale manufacturing.

## 2. Fabrication Process

**Figure 1:**
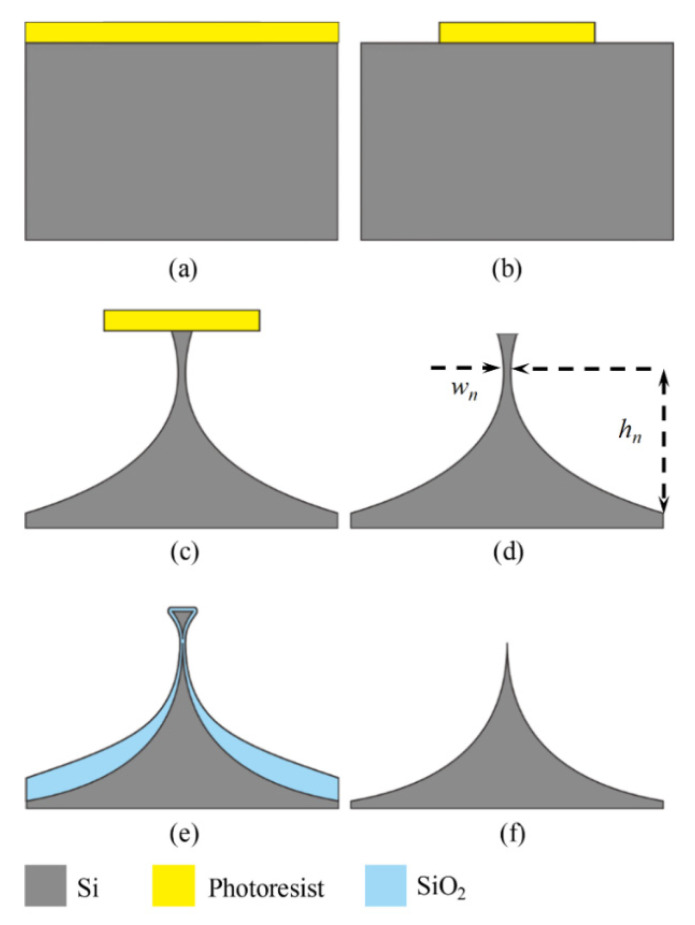
Fabrication process flow for silicon tips. (**a**) photoresist layer as etching mask, (**b**) patterning the photoresist layer, (**c**) isotropic etching of silicon, (**d**) removal of the mask, (**e**) thermal oxidation, (**f**) removal of the oxide layer.

Figure 1 described the fabrication process of a nanotip based on isotropic ICP etching, including patterning the photoresist, isotropic etching of silicon, tip sharpening by oxidation [41], and tip releasing. A 4-inch (100)-oriented Si wafer was used (IceMOS, Hannahstown, Belfast, UK). The lithography was performed to define the tip apex, and the silicon substrate was isotropically dry etched using Advanced Silicon Etch from Oxford (Oxford Instruments, Abingdon, Oxon, UK) to a depth of 16 μm, finally, a neck was formed, as illustrated in Figure 1b. 

The Si isotropic etching process is critical in producing pre-tips with small neck width *w_n_*, large neck height *h_n_*, and small surface roughness. For making a tip with height greater than 10 μm, aspect ratio greater than 3:1, and apex radius smaller than 5 nm, pre-tip sharpening process by oxidation also needs to be modified [42]. After removal of oxide layer by buffered hydrogen fluoride (BHF), the tip height and diameter were examined by scanning electron microscope (SEM) (FEI, Hillsboro, OR, USA).

## 3. Results and Discussion 

**Figure 2:**
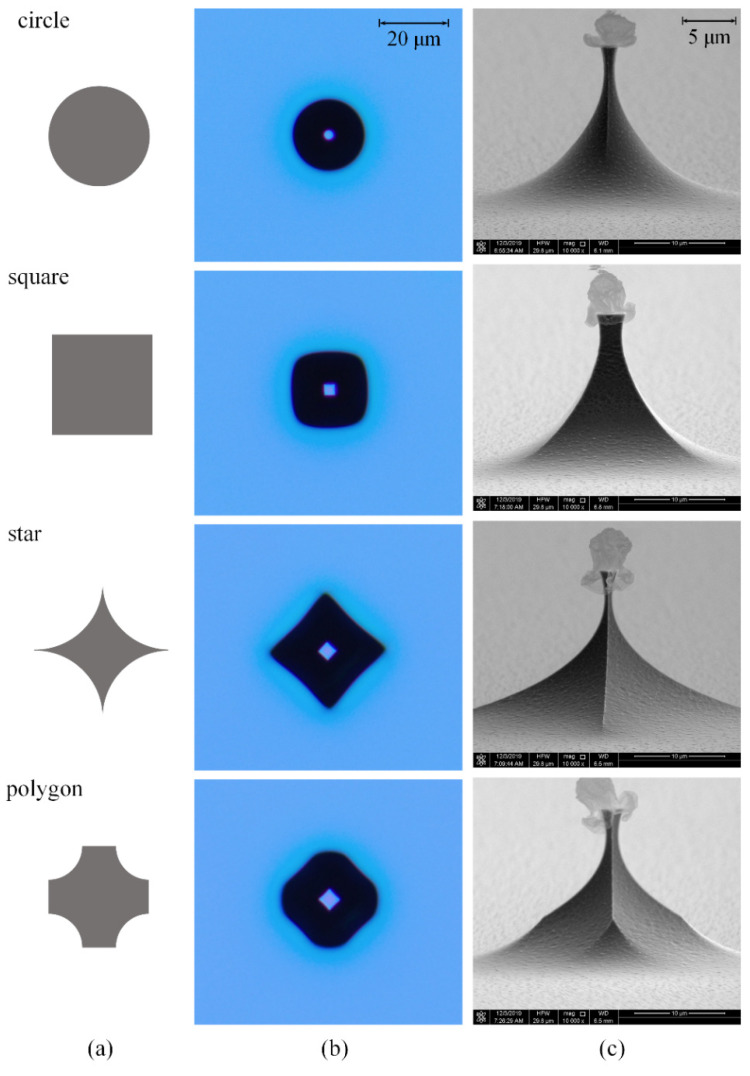
Different tip masks (**a**) and corresponding pre-tips after etching with the same recipe: pressure is 9 mTorr, flow rate is 30 sccm, inductively coupled plasma (ICP) power is 1000 W and platen power is 0 W. (**b**) are top view images and (**c**) are scanning electron microscope (SEM) images.

The shapes of the pre-tips made by isotropic ICP etching, such as the height and aspect ratio, are influenced by the mask design, which affects the gas supply and venting of reaction products of the pre-tip. Moreover, the profiles of the tips made with the conventional aperture mask and the “island mask” show clear differences [43]. The mask pattern shown in Figure 2a was designed to investigate the effect of gas supply from different directions during the etching process.

The round and square masks can realize large tip height and better tip geometry. As shown in Figure 2b, the star and polygon masks are apt to gather SF_6_ gas, cause more reflection of fluorine radicals between the bottom and sidewall, and decrease the neck height. The normalized tip height, the ratio of the tip height to the etch depth, and the normalized neck width, the ratio of the neck width of pre-tip to the mask size, are used to evaluate the etched pre-tip profile. The normalized tip heights and neck width for different masks are summarized in Table 1. Compared with the tips with a normalized height from 0.7 to 0.8 made by wet etching [26], the tips produced by dry etching with circle and square mask have larger normalized heights.

**Figure 3:**
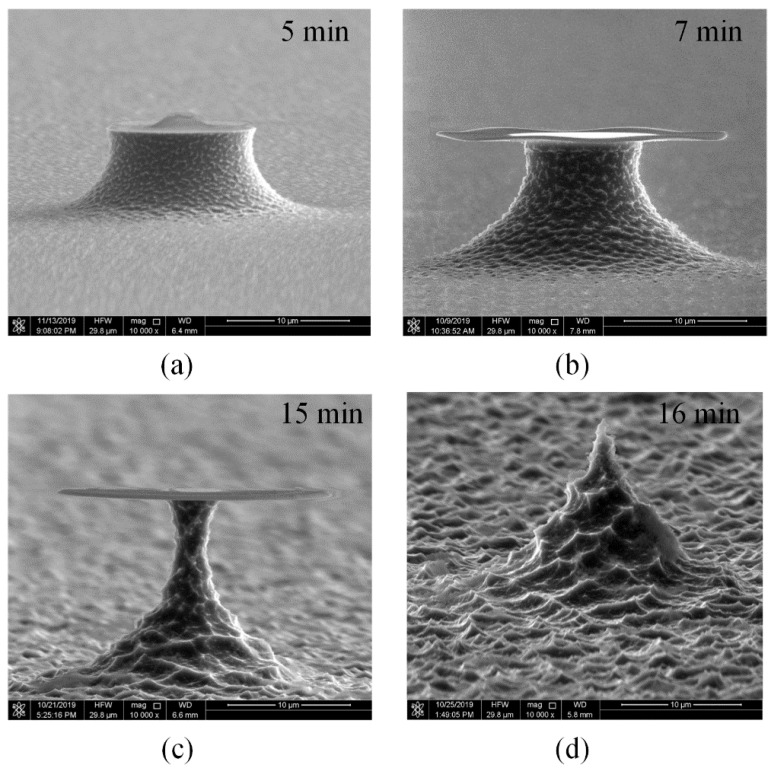
The tip profiles after etching for different duration with the same recipe: pressure is 3 mTorr, flow rate is 20 sccm, ICP power is 1000 W and platen power is 0 W. (**a**) 5 min, (**b**) 7 min, (**c**) 15 min, (**d**) 16 min.

For achieving a large tip height (greater than 10 μm), it is needed to increase the mask size and the etch depth. However, the long etching time will greatly deteriorate the surface roughness [44], as shown in Figure 3d, the tip neck could randomly break off and it is hard to make the nanometer tip apex [36].

The tip profiles strongly depend on the etching condition, such as chamber pressure, gas flow rate, the ICP power, and the platen power. The recipe of pure SF_6_ isotropic etching are illustrated in Table 2, the ICP power are kept constant to avoid its effect on the roughness of the etched surface [44].

**Figure 4:**
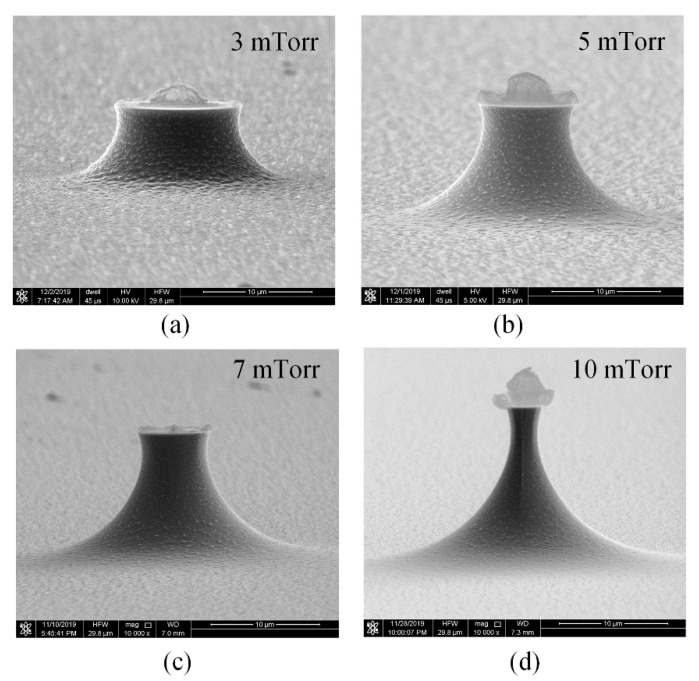
The tip profiles etched with circular masks under different chamber pressures with 30 sccm flow rate, 1000 W ICP power and 0 W platen power. (**a**) 3 mTorr, (**b**) 5 mTorr, (**c**) 7 mTorr, (**d**) 10 mTorr.

Figure 4 shows the tip profiles etched with circular masks under different chamber pressures, the tip surface roughness is greatly improved with enhanced pressure. Tip masks on the wafer are a kind of “island mask”, thus tip etching is different from the common cavity etching. The tip profiles are more dependent on the chemical reaction than venting of the exhausting gas. Under a low chamber pressure, there are no enough fluorine radicals to reach Si surface, thus the etching rate from point to point of the Si tip are not uniform, and results in large surface roughness. Increasing the pressure can supply more fluorine radicals to Si surface and ensure uniform reaction and achieve small surface roughness. Therefore, increasing the chamber pressure could effectively reduce the surface roughness.

**Figure 5:**
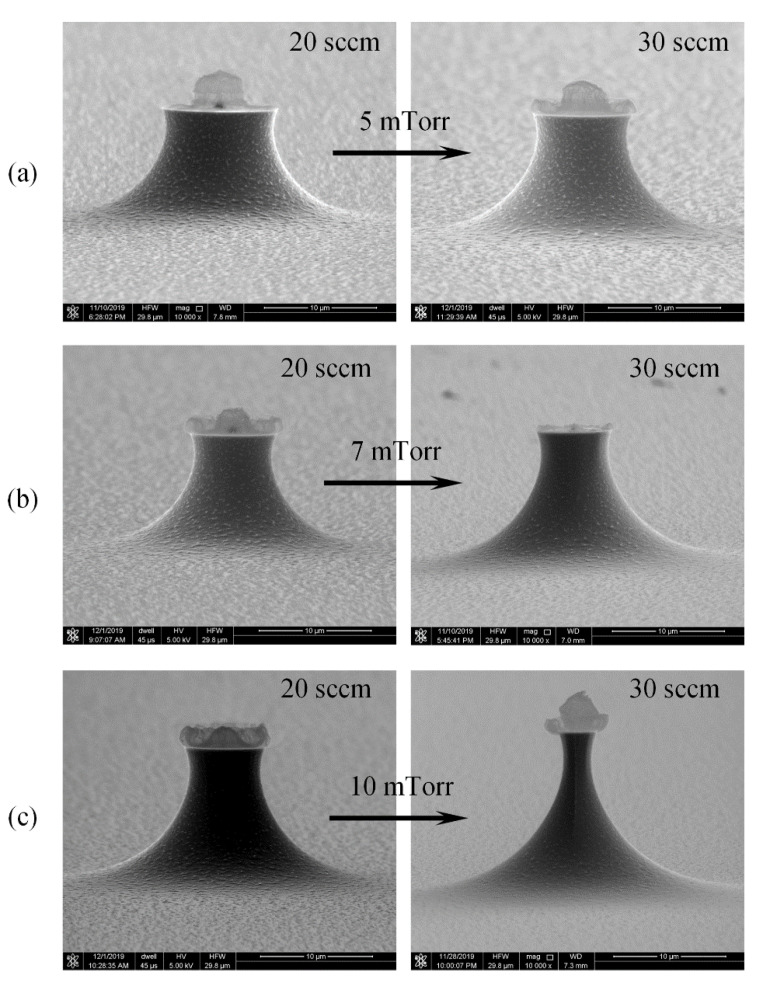
Dependence of tip profile and roughness on SF 6 gas flow rate with 1000 W ICP power and 0 W platen power. (**a**) 5 mTorr, (**b**) 7 mTorr, (**c**) 10 mTorr.

Figure 5 depicts the effects of SF_6_ flow rates on the tip profiles etched under different chamber pressures. For low chamber pressures of 5 mTorr and 7 mTorr, the roughness had no clear change when the SF_6_ flows rates increased 50%, since in low chamber pressures, venting of the reaction product surpasses SF_6_ gas supply, no more chemical reaction takes place around the mask even at higher gas flow rate, thus the surface roughness was almost unchanged.

When the chamber pressure goes up to 10 mTorr, venting process of the exhausting product slows down, the high gas flow rate can supply enough SF_6_ for chemical reaction, thus the surface roughness was dramatically improved. 

The etching rate is determined by thermal, physical, and ion-assisted etching [45]:

(1) $$ER_{total}=ER_{thermal}+ER_{physical}+ER_{ionassisted}$$

where *ER_total_* is the total etching rate, *ER_thermal_* is the spontaneous etching of silicon by fluorine atoms in the absence of ion bombardment. *ER_physical_* is the physical sputteriing of surface atoms by energetic alone. And *ER_ion assisted_* accounts for the greatly enhanced etching during simultaneous reactant and ion exposure.

Isotropic etching of Si by SF_6_ is a chemical process, the etch rate of silicon by fluorine atoms can be estimated by [45]:

(2) $$ER_{total}=k_{0}\cdot Q_{F}\cdot \exp\left(\frac{-E_{a}}{k_{b}\cdot T}\right)$$

where *k_0_* and *k_b_* are constants, *Q_F_* is the flux of the fluorine atoms, *E_a_* is the activation energy, and *T* is the absolute temperature. At constant temperature, the etch rate is proportional to *Q_F_*. When the flow rate is too high, it is difficult to precisely control the neck width. Therefore, the gas flow rate and the chamber pressure should be optimized for making the pre-tip with large height and small roughness.

**Figure 6:**
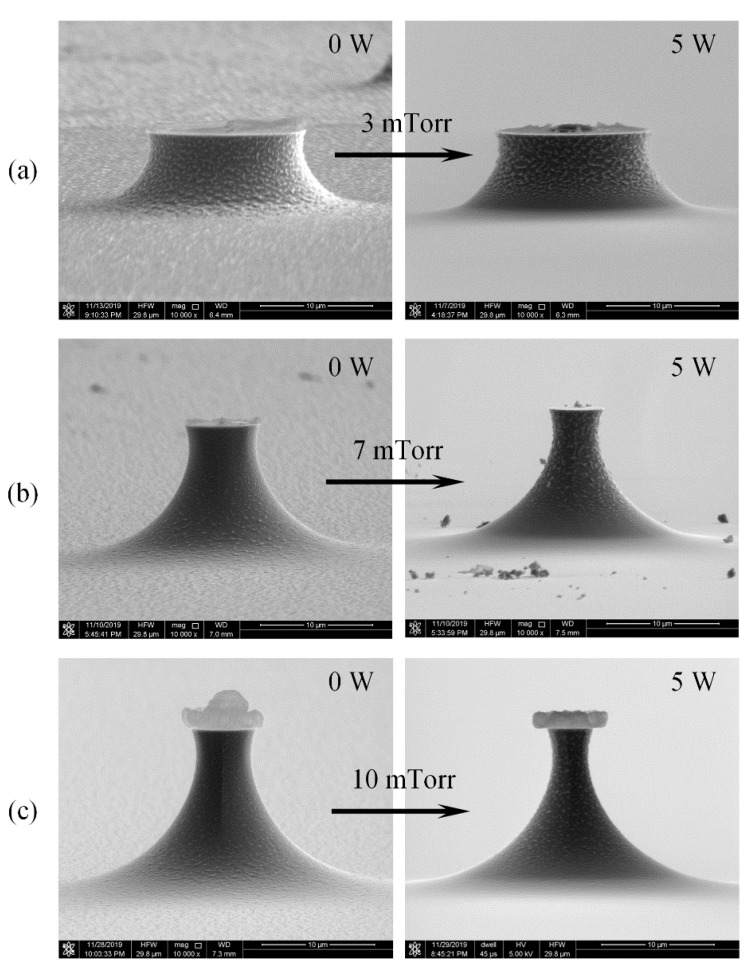
Tip profile fabricated with different platen power with 20 sccm flow rate and 1000 W ICP power. (**a**) 3 mTorr, (**b**) 7 mTorr, (**c**) 10 mTorr.

In addition, applying a radio frequency platen power accelerates fluorine radicals vertically towards the Si wafer. The density of fluorine radicals reaching the bottom of Si tip increases, the ion energy rises and the tip bottom is etched mainly by ion bombardment, the bottom roughness is reduced. Meanwhile, the ion bombardment to the tip sidewall under the mask is clearly reduced, and the density of fluorine radicals reaching the sidewall of the tip decreases, the chemical reaction slow down and the surface roughness increase. As shown in Figure 6, increasing the platen power lead to a dramatic improvement on the surface roughness, especially the bottom surface. However, with a constant SF_6_ flow rate, the increase in gas density on the bottom surface resulted in the reduced gas supply to the sidewall surface and eventual rough side wall, which will be apt to a larger tip radius of curvature. Hence, the platen power should also be optimized. 

When the chamber pressure is optimized to 9 mTorr, the SF_6_ gas flow is 30 sccm, the ICP power is 1000 W, and the platen power is 0 W, the pre-tip with large height and small surface roughness can be obtained.

**Figure 7:**
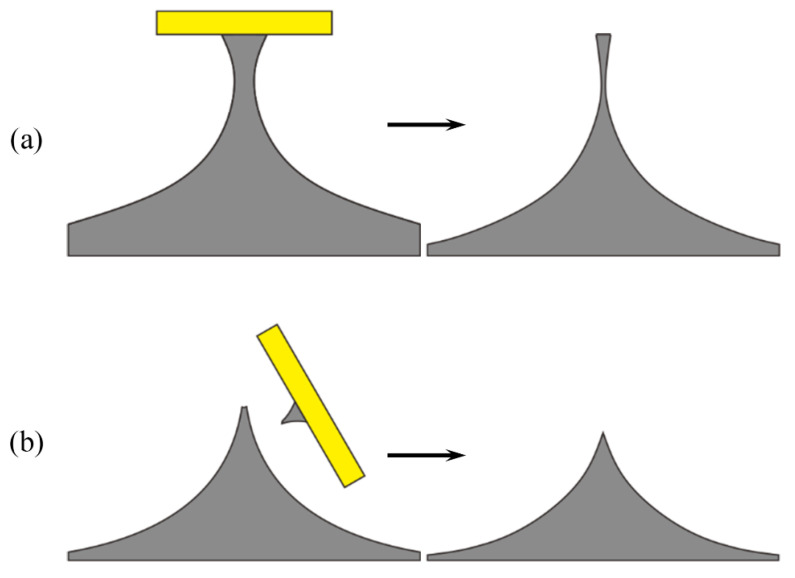
Tip profile after isotropic insufficient etching (**a**) and over-etching (**b**) as well as thermal oxidation.

The isotropic etching time is important for achieving high aspect ratio of the pre-tip. As shown in Figure 7a, insufficient etching usually leads to large neck widths and eventual large tip apexes after oxidation sharpening, over-etching could result in very small neck widths, which are too fragile to support the tip mask, and the heights and aspect ratios of the tips are greatly reduced after oxidation.

Besides, thermal oxidation of Si at low-temperature for tip sharpening is limited by the oxide thickness, usually less than 500 nm, thus the neck width should be less than 500 nm [41]. Therefore, precisely controlling the isotropic etching rate and time is critical for realizing a desired neck width within a tolerance as small as tens of nanometers. 

The isotropic etching volume per unit time can be expressed as [44]:

(3) $$\frac{d{\tilde{V}}_{etch}}{d\tilde{t}}=P_{etch}$$

where $d{\tilde{V}}_{etch}$ and $d\tilde{t}$ are the dimensionless normalized etching volume and dimensionless normalized etching time, respectively. *P_etch_* is the probability that the fluorine radical is consumed during the etching process, which is related to both the geometry of the etched cavity and the surface sticking coefficient of the radicals. 

For the “island mask” formed in tip etching process, the etched cavity is approximately infinite and the fluorine radicals completely react, so *P_etch_* can be considered as constant. 

In isotropic etching, the removed volume can be decomposed into vertical etching volume and horizontal etching volume for all cross section per unit time, which can be expressed as:

(4) $$V_{etch}=\int_{0}^{H}\left(ER_{ver}+ER_{hor}\right)\cdot \Delta t\cdot dh$$

where *H* is the etch depth, *ER_ver_* and *ER_hor_* are the surface etching rates in vertical and horizontal directions, respectively, with the units of μm^2^/s. *Δt* is the unit etching time. For a certain mask, the vertical surface etching rate is constant. 

For any etching cross section in the horizontal direction, the etching rate can be experimentally obtained, thus the etching time needed for a desired neck width of the pre-tip can be estimated by:

(5) $$S_{1}-S_{0}=ER_{hor}\cdot \Delta t$$

where *S_1_* and *S_0_* are the area of the pre-tip cross-sections along the horizontal direction before and after etching, respectively. 

**Figure 8:**
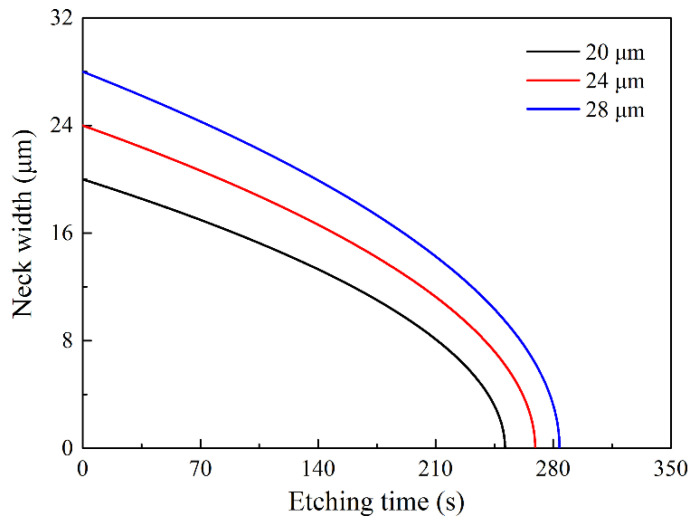
Dependence of the neck widths on the etching time for the circular masks with different sizes.

Taking a circular mask as an example, the neck width can be estimated with Equation (4):

(6) $$\frac{\pi }{4}\left(w_{1}^{2}-w_{2}^{2}\right)=ER_{hor}\cdot \Delta t$$

where *w_1_* and *w_0_* are the neck widths before and after the etching time, respectively. The relationship between the neck width and the etching time for different mask sizes are given in Figure 8.

**Figure 9:**
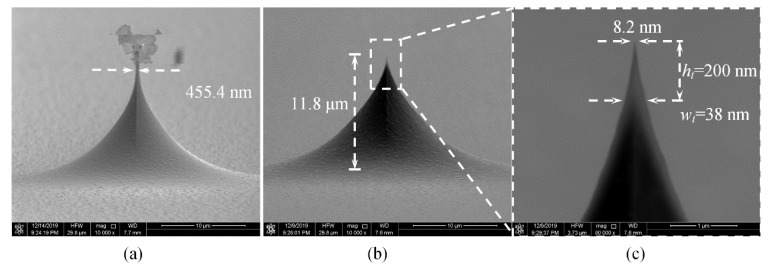
SEM images of pre-tip (**a**) and tip after oxidation sharpening (**b**,**c**).

For a circular mask with a diameter of 24 μm, after etching for 266 s, the calculated neck width is 500 nm. As shown in Figure 9a, the etched neck width is 455.4 nm, close to the expected value. The tip after oxidation sharpening has a height of 11.8 μm, the radius curvature of 4.1 nm, and the aspect ratio of 5.2:1 @ 200 nm which represents the ratio of *h_t_* to *w_t_* while the tip height reaches 200 nm, as demonstrated in Figure 9c. This process uses only one mask, is very simple compared to the multi-step etching [39].

**Figure 10:**
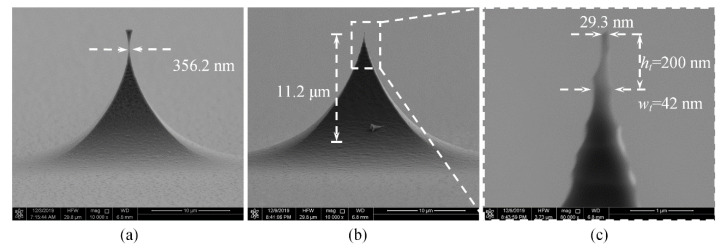
SEM images of pre-tip (**a**) and tip after oxidation sharpening (**b**,**c**).

For a square mask with a length of 24 μm, after etching for 243 s, the calculated neck width is 430 nm. As shown in Figure 10, the etched neck width is 356.2 nm. The tip after oxidation sharpening has a height of 11.2 μm, the radius curvature of 14.7 nm, and the aspect ratio of 4.7: 1 at a tip height of 200 nm. Compared to the circular mask, the square mask results in a rougher tip surface, as shown in Figure 10c, greatly reducing the radius curvature and aspect ratio.

For wafer-level fabrication, the etching gas is unevenly distributed around the wafer, and this results in the “edge effect”, that is, the Si etching rates in the central region are smaller than those at wafer edge [46,47], thus a great variation of the neck widths at wafer level occurs. Therefore, it is necessary to compensate the tip mask along the wafer in order to improve the etching uniformity. 

Firstly, a “dummy mask” is designed for the compensation of tip mask to reduce etching area and optimize the exposure ratio from 75% to 60%. Secondly, various compensation patterns are designed for different locations of the wafer to further balance the etching area and gradually reduce the exposure ratio from the wafer edge to the center. The wafer-level etching uniformity is shown in Table 3:

(7) $$Non-uniformity=2\frac{D_{\max}-D_{\min}}{D_{\max}+D_{\min}}$$

where *D_max_* is the maximum etching depth and *D_min_* is the minimum etching depth along the wafer. 

In case of no compensation, the exposure ratio is as high as 75%, and the non-uniformity is 9.4%. Adding a “dummy mask” to the layout, the uniformity is improved. Finally, with full compensation, the non-uniformity can be reduced to 0.3%, which greatly improves the yield of nano tips. According to the optimized etching recipe, etching time and mask pattern in this work, tips were batch fabricated on wafer-scale and the fabricated tips with apex radius less than 5 nm, aspect ratio greater than 5 at a tip height of 200 nm, and tip height more than 10 μm were produced with high fabrication yield up to 95%. 

## 4. Conclusions

This work presents a novel batch fabrication approach for Si nanometer tips based on isotropic ICP etching technology, where the etching non-uniformity at a wafer-level is controlled within 0.3%. By mask compensation and precisely controlling the lateral etch depth, the silicon tips with apex radius less than 5 nm, aspect ratio greater than 5 at a tip height of 200 nm, and tip height more than 10 μm were produced with high fabrication yield. This fabrication process is simple and reliable and has potential application in the development of high-end nanometer Si tip-based devices. 

## Figures and Tables

**Table 1:** Normalized tip heights for different masks.

Mask Shape	Normalized Tip Height (Etching for 5 min)	Normalized Neck Width (Etching for 5 min)
Circle	0.82	0.018
Square	0.80	0.026
Star	0.71	0.027
polygon	0.67	0.039

**Table 2:** Etching parameters.

Pressure (mTorr)	Flow Rate (sccm)	ICP Power and Frequency (W/MHz)	Platen Power and Frequency (W/MHz)	Etching Time (min)
3 3 5 5 7 7 7 9 9 10 10 10	20 20 20 30 20 30 30 30 30 20 30 30	1000/2 1000/2 1000/2 1000/2 1000/2 1000/2 1000/2 1000/2 1000/2 1000/2 1000/2 1000/2	0 5/13.56 0 0 0 0 5/13.56 0 5/13.56 0 0 5/13.56	5 5 5 5 5 5 5 5 5 5 5 5

**Table 3:** Non-uniformity achieved with different compensation modes.

Compensation Mode	Non-Uniformity (%)
No Compensation	9.4
Partial Compensation	6.6
Full Compensation	0.3

## References

[1] Lucas M., Gall K., Riedo E. (2008). Tip size effects on atomic force microscopy nanoindentation of a gold single crystal. J. Appl. Phys..

[2] Wang F., Li X., Guo N., Wang Y., Feng S. (2006). A silicon cantilever probe card with tip-to-pad electric feed-through and automatic isolation of the metal coating. J. Micromech. Microeng..

[3] Wang F., Li X., Cheng R., Jiang K., Feng S. (2009). Silicon cantilever arrays with by-pass metal through-silicon-via (TSV) tips for micromachined IC testing probe cards. Microelectron. Eng..

[4] Olfat M., Strathearn D., Lee G., Sarkar N., Hung S.C., Mansour R.R. A single-chip scanning probe microscope array. Proceedings of the IEEE International Conference on Micro Electro Mechanical Systems.

[5] Sun K., Zhang W., Li B., Lee J.Y., Xie Y.-H., Schroeder T., Katzer J., Wei X., Russell T.P. (2012). Field Emission Tip Array Fabrication Utilizing Geometrical Hindrance in the Oxidation of Si. IEEE Trans. Nanotechnol..

[6] Resnik D., Možek M., Pečar B., Janež A., Urbančič V., Iliescu C., Vrtačnik D. (2018). In Vivo Experimental Study of Noninvasive Insulin Microinjection through Hollow Si Microneedle Array. Micromachines.

[7] Folch A., Wrighton M.S., Schmidt M.A. (1997). Microfabrication of oxidation-sharpened silicon tips on silicon nitride cantilevers for atomic force microscopy. J. Microelectromech. Syst..

[8] Grow R., Minne S., Manalis S., Quate C. (2002). Silicon nitride cantilevers with oxidation-sharpened silicon tips for atomic force microscopy. J. Microelectromech. Syst..

[9] Kaushik S., Hord A.H., Denson D.D., McAllister D.V., Smitra S., Allen M.G., Prausnitz M.R. (2001). Lack of Pain Associated with Microfabricated Microneedles. Anesth. Analg..

[10] Kim Y.C., Park J.H., Prausnitz M.R. (2012). Microneedles for drug and vaccine delivery. Adv. Drug Deliv. Rev..

[11] Mamin H.J., Ried R., Terris B., Rugar D. (1999). High-density data storage based on the atomic force microscope. Proc. IEEE.

[12] Persaud A., Park S.J., Liddle J.A., Schenkel T., Bokor J., Rangelow I.W. (2005). Integration of Scanning Probes and Ion Beams. Nano Lett..

[13] Costa M., Tarequzzaman M., Ferreira R., Cardoso S., Gaspar J., Cardoso S.F. Towards high-resolution scanning magnetoresistance microscopy. Proceedings of the 2017 IEEE 12th International Conference on Nano/Micro Engineered and Molecular Systems (NEMS).

[14] Itoh H., Odaka T., Niitsuma J. (2014). SPM system for semiconductor device applications. Microscopy.

[15] Myhra S. (2004). Manipulation of Si oxide and electrically conducting carbon films by scanning probe microscopy (SPM): Nano-lithography and nano-machining. Thin Solid Films.

[16] Lee S.H., Lim G., Moon W. (2007). A Novel SPM Probe with MOS Transistor and Nano Tip for Surface Electric Properties. J. Phys. Conf. Ser..

[17] Rodriguez B.J., Jesse S., Seal K., Baddorf A.P., Kalinin S.V., Rack P.D. (2007). Fabrication, dynamics, and electrical properties of insulated scanning probe microscopy probes for electrical and electromechanical imaging in liquids. Appl. Phys. Lett..

[18] University of Pittsburgh, Stanford University (2005). Selective Growth of Si Nanowire Arrays Via Galvanic Displacement Processes in Water-in-Oil Microemulsions. J. Am. Chem. Soc..

[19] Qian W., Sun S., Song J., Nguyen C., Ducharme S., Turner J.A. (2018). Focused electron-beam-induced deposition for fabrication of highly durable and sensitive metallic AFM-IR probes. Nanotechnology.

[20] Shandyba N.A., Kolomiytsev A.S., Panchenko I.V., Lisitsyn S.A. (2019). Novel technology for fabrication of probe tips for SPM using focused ion beam-induced deposition method. IOP Conf. Ser. Mater. Sci. Eng..

[21] Givargizov M.E., Stepanova A.N., Obolenskaya L.N. (2003). Technology “WhiskerProbes”. AIP Conference Proceedings.

[22] Campanella H., Jaafar M., Llobet J., Esteve J., Vazquez M., Asenjo A., Del Real R.P., Plaza J.A. (2011). Nanomagnets with high shape anisotropy and strong crystalline anisotropy: Perspectives on magnetic force microscopy. Nanotechnology.

[23] Menozzi C., Gazzadi G.C., Alessandrini A., Facci P. (2005). Focused ion beam-nanomachined probes for improved electric force microscopy. Ultramicroscopy.

[24] Yapici M.K., Zou J. (2008). A novel micromachining technique for the batch fabrication of scanning probe arrays with precisely defined tip contact areas. J. Micromech. Microeng..

[25] Chang W.S., Jeong M.S., Kim D.C., Kim J. (2007). Fabrication of Cantilevered Tip-on-Aperture Probe for Enhancing Resolution of Scanning Near-Field Optical Microscopy System. Jpn. J. Appl. Phys..

[26] Tang B., Sato K., Gosálvez M.A. (2012). Sharp silicon tips with different aspect ratios in wet etching/DRIE and surfactant-modified TMAH etching. Sens. Actuators A.

[27] Bhandari R., Negi S., Rieth L., Solzbacher F. (2010). A wafer-scale etching technique for high aspect ratio implantable MEMS structures. Sens. Actuators, A.

[28] Zhang X., Yu X., Li T., Wang Y. (2018). A novel method to fabricate silicon nanoprobe array with ultra-sharp tip on (111) silicon wafer. Microsyst. Technol..

[29] Imaeda K., Bessho K., Shikida M. (2016). Design and fabrication of differently shaped pyramids on Si{100} by anisotropic wet etching. Microsyst. Technol..

[30] Izumi H., Okamoto T., Suzuki M., Aoyagi S. (2009). Development of a Silicon Microneedle with Three-Dimensional Sharp Tip by Electrochemical Etching. IEEJ Trans. Sens. Micromach..

[31] Hamzah A.A., Aziz N.A., Majlis B.Y., Yunas J., Dee C.F., Bais B. (2012). Optimization of HNA etching parameters to produce high aspect ratio solid silicon microneedles. J. Micromech. Microeng..

[32] Carta S., Bagni R., Giovine E., Foglietti V., Evangelisti F., Notargiacomo A. (2013). Fabrication of bulk and epitaxial germanium field emitter arrays by dry etching techniques. Microelectron. Eng..

[33] Chen K.J., Fang T.H., Ji L.W., Chang S.J., Young S.J. (2014). Fabrication and characteristics of silicon micro-tip arrays. Int. J. Mod. Phys. B.

[34] Boisen A., Hansen O., Bouwstra S. (1996). AFM probes with directly fabricated tips. J. Micromech. Microeng..

[35] Rakhshandehroo M.R. (1996). Simulation and dry etching of field emitter tips in Si. J. Vac. Sci. Technol. A Vac. Surf. Films.

[36] Shin Y.M., Kim Y.K., Lee S.K., Park J.H. (2018). Single-mask fabrication of micro-probe electrode array with various tip heights and sharpness using isotropic and anisotropic etching. Micro Nano Lett..

[37] Villanueva G., Plaza J.A., Sanchez A., Zinoviev K., Perez-Murano F., Bausells J. (2007). DRIE based novel technique for AFM probes fabrication. Microelectron. Eng..

[38] Held J., Gaspar J., Ruther P., Hagner M., Cismak A., Heilmann A., Paul O. (2010). Design of experiment characterization of microneedle fabrication processes based on dry silicon etching. J. Micromech. Microeng..

[39] Kanechika M., Mitsushima Y. (2000). Silicon Needles Fabricated by Highly Selective Anisotropic Dry Etching and Their Field Emission Current Characteristics. Jpn. J. Appl. Phys..

[40] Kim D.W., Lym S.H., Jung M.Y. (1999). Fabrication of field emission Si-tip array using reduced submicron masks generated by isotropic etching of mask patterns. Microelectron. Eng..

[41] He H., Zhang J., Yang J., Yang F. (2016). Silicon tip sharpening based on thermal oxidation technology. Microsyst. Technol..

[42] Im H., Oh S.H. (2014). Oxidation Sharpening, Template Stripping, and Passivation of Ultra-Sharp Metallic Pyramids and Wedges. Small.

[43] Panduranga P., Abdou A., Ren Z., Pedersen R.H., Nezhad M. (2019). Isotropic silicon etch characteristics in a purely inductively coupled SF6 plasma. J. Vac. Sci. Technol. B.

[44] Larsen K.P., Petersen D.H., Hansen O. (2006). Study of the Roughness in a Photoresist Masked, Isotropic, SF_6_-Based ICP Silicon Etch. J. Electrochem. Soc..

[45] Arnold J.C. (1993). Influence of reactant transport on fluorine reactive ion etching of deep trenches in silicon. J. Vac. Sci. Technol. B Microelectron. Nanometer Struct..

[46] Abe H., Yoneda M., Fujiwara N. (2008). Developments of Plasma Etching Technology for Fabricating Semiconductor Devices. Jpn. J. Appl. Phys..

[47] Donnelly V.M., Kornblit A. (2013). Plasma etching: Yesterday, today, and tomorrow. J. Vac. Sci. Technol. A Vac. Surf. Films.

